# Comment on: Fetal femur length and risk of diabetes in adolescence: a prospective cohort study

**DOI:** 10.1186/s41182-024-00627-y

**Published:** 2024-10-30

**Authors:** Zainab Fatima, Arifa Inayatullah Kakar, Usama Idrees, Malik Olatunde Oduoye, Uzodinma Nwadinigwe

**Affiliations:** 1 Shaikh Khalifa Bin Zayed Al Nahyan Medical and Dental College, Lahore, Pakistan; 2 Dow University of Health Sciences (DUHS), V256+282, Mission Rd, Nanak Wara Nanakwara, Karachi City, Sindh Pakistan; 3 Khawaja Muhammad Safdar Medical College, Sialkot, Pakistan; 4 Department of Research, The Medical Research Circle (MedReC), PO Box73, Goma, Democratic Republic of the Congo; 5 University of Nigeria Teaching Hospital, Enugu, Nigeria

**Keywords:** Fetal femur length, Diabetes in adolescence, Gestational diabetes mellitus, Type 2 diabetes mellitus, Genetic predisposition

## Abstract

We read the article "Fetal Femur Length and Risk of Diabetes in Adolescence: A Prospective Cohort Study" by Sayeed et al. with great interest. The authors present compelling evidence linking mid-trimester impaired femur growth with elevated prediabetic biomarkers in Bangladeshi adolescents. However, we believe the study would benefit from considering additional factors. Maternal gestational diabetes, a well-known risk factor for type 2 diabetes mellitus (T2DM) in offspring, and family history of diabetes, which reflects genetic predisposition, should be included. Socioeconomic factors, which influence health outcomes, also warrant attention. Including these variables would provide a more comprehensive understanding of the relationship between fetal femur length and T2DM risk in adolescents.

**Dear Editor,**

We have read with sincerity the article “Fetal femur length and Risk of Diabetes in Adolescence: A Prospective Cohort Study” by Sayeed et al. [1]. The authors did their best to craft a concisely written article and have described the scenarios amazingly, leading to agreement with the conclusion that mid-trimester impaired femur growth may be associated with elevated prediabetic biomarkers in Bangladeshi adolescents. However, we would like to mention a few postulates that would enhance the quality of the article. Firstly, as a predisposing factor in the development of diabetes mellitus (DM) in adolescents, mothers’ records of gestational DM should also be considered. Type 2 diabetes mellitus (T2DM) development in offspring is strongly associated with the maternal diabetic intrauterine environment, which is evident in a multiethnic population in which mothers of 30.4% of youth with T2DM had a history of DM in comparison to mothers of 6.3% of non-diabetic youth controls [2].

Secondly, genetics play a pivotal role in developing adolescent diabetes; therefore, family history should be considered [2, 3]. In addition to socioeconomic factors [3], a study by Reinehr et al. revealed that chronic inflammation might be a causative agent of the development of T2DM. Certain inflammatory markers can be prognostic markers for T2DM, such as elevated levels of TNF alpha and IL6, which are found before T2DM onset, as in gestational diabetes [4]. Thirdly, overlooking gestational diabetes as a determinant for decreased fetal femur length and gradual development of T2DM in youth has serious implications, as a strong link has been found between gestational diabetes, fetal growth patterns and a higher risk of T2DM in babies. Mothers’ HbA1c levels should also be monitored as it could help establish a true association between fetal femur length and the likelihood of T2DM development in young adults [5]. Lastly, a lack of physical activity and consumption of caffeinated drinks are a significant known risk factor for T2DM, especially in rural areas like Matlab where this cohort was conducted. Therefore, physical inactivity and caffeinated beverage intake should also be taken into account as a major risk factor for T2DM [3].

In short, the article by Sayeed et al. provides key insights into the association between fetal femur length and T2DM incidence in youth [1]. However, a broader perspective could be attained by taking into account the effect of gestational diabetes, socioeconomic factors, and family history on DM and fetal femur length.

## Data Availability

Not applicable.
