# Correction: Comment on: Fetal femur length and risk of diabetes in adolescence: a prospective cohort study

**DOI:** 10.1186/s41182-024-00667-4

**Published:** 2024-12-19

**Authors:** Zainab Fatima, Arifa Inayatullah Kakar, Usama Idrees, Malik Olatunde Oduoye, Uzodinma Nwadinigwe

**Affiliations:** 1 Shaikh Khalifa Bin Zayed Al Nahyan Medical and Dental College, Lahore, Pakistan; 2 Dow University of Health Sciences (DUHS), V256+282, Mission Rd, Nanak Wara Nanakwara, Karachi City, Sindh Pakistan; 3 Khawaja Muhammad Safdar Medical College, Sialkot, Pakistan; 4 Department of Research, The Medical Research Circle (MedReC), PO Box73, Goma, Democratic Republic of the Congo; 5 University of Nigeria Teaching Hospital, Enugu, Nigeria

**Correction: Tropical Medicine and Health (2024) 52:76** 10.1186/s41182-024-00627-y

Following publication of the original article [1], the authors reported that the second author’s name needed to be changed from “Arifa Inayatullah” to “Arifa Inayatullah Kakar”, and the first and third affiliations needed to be updated.

The incorrect affiliations were:

^1^Shaikh Khalifa Bin Zayed Al-Nahyan Medical and Dental College, G855+XRM, Sheikh Zayed Medical Complex Khayaban-E-Jamia Punjab, Block D Muslim Town, Lahore, Punjab, India.

^3^Khawaja Muhammad Safdar Medical College, GH32+J2G, Sialkot, Punjab, India.

The correct affiliations are:

^1^Shaikh Khalifa Bin Zayed Al Nahyan Medical and Dental College, Lahore, Pakistan.

^3^Khawaja Muhammad Safdar Medical College, Sialkot, Pakistan.

The correct affiliations have been provided in this correction. The original article [1] has been corrected.

## References

[1] Fatima Z, Kakar AI, Idrees U, Oduoye MO, Nwadinigwe U. Comment on: Fetal femur length and risk of diabetes in adolescence: a prospective cohort study. Trop Med Health. 2024;52:76. 10.1186/s41182-024-00627-y. 39478613 10.1186/s41182-024-00627-y PMC11523857

